# Building a Scaffold for Arteriovenous Fistula Maturation: Unravelling the Role of the Extracellular Matrix

**DOI:** 10.3390/ijms241310825

**Published:** 2023-06-28

**Authors:** Suzanne L. Laboyrie, Margreet R. de Vries, Roel Bijkerk, Joris I. Rotmans

**Affiliations:** 1 Department of Internal Medicine, Leiden University Medical Centre, 2333 ZA Leiden, The Netherlands; s.l.laboyrie@lumc.nl (S.L.L.); r.bijkerk@lumc.nl (R.B.); 2 Department of Surgery and the Heart and Vascular Center, Brigham & Women’s Hospital and Harvard Medical School, Boston, MA 02115, USA; m.r.de_vries@lumc.nl; 3 Department of Vascular Surgery, Leiden University Medical Centre, 2333 ZA Leiden, The Netherlands

**Keywords:** extracellular matrix, arteriovenous fistula, vascular remodelling, AVF maturation

## Abstract

Vascular access is the lifeline for patients receiving haemodialysis as kidney replacement therapy. As a surgically created arteriovenous fistula (AVF) provides a high-flow conduit suitable for cannulation, it remains the vascular access of choice. In order to use an AVF successfully, the luminal diameter and the vessel wall of the venous outflow tract have to increase. This process is referred to as AVF maturation. AVF non-maturation is an important limitation of AVFs that contributes to their poor primary patency rates. To date, there is no clear overview of the overall role of the extracellular matrix (ECM) in AVF maturation. The ECM is essential for vascular functioning, as it provides structural and mechanical strength and communicates with vascular cells to regulate their differentiation and proliferation. Thus, the ECM is involved in multiple processes that regulate AVF maturation, and it is essential to study its anatomy and vascular response to AVF surgery to define therapeutic targets to improve AVF maturation. In this review, we discuss the composition of both the arterial and venous ECM and its incorporation in the three vessel layers: the tunica intima, media, and adventitia. Furthermore, we examine the effect of chronic kidney failure on the vasculature, the timing of ECM remodelling post-AVF surgery, and current ECM interventions to improve AVF maturation. Lastly, the suitability of ECM interventions as a therapeutic target for AVF maturation will be discussed.

## 1. Introduction

End-stage kidney disease (ESKD) patients receive renal replacement therapy through kidney transplantation or dialysis treatment. During haemodialysis (HD), vascular access (VA) connects the patient’s blood supply to the dialysis machine. VA can be achieved through a central venous catheter (CVC), arteriovenous graft (AVG), or an arteriovenous fistula (AVF). The radiocephalic AVF was the first arteriovenous configuration, introduced in 1966 [1]. This provided a stepping stone for a high-flow vascular access design for HD, served as inspiration for other AVF configurations, and resulted in the AVF being the gold standard in VA [2].

The National Kidney Foundation’s Kidney Disease Outcomes Quality Initiative (KDOQI) guidelines recommend AV access (AVF or AVG) in patients requiring HD [3]. AVFs have better longevity and reduced complications when compared to AVG and CVC [4,5,6,7]. However, non-maturation rates are around 9–31%, depending on AVF type, compared to 9% functional failure in AVGs, which remains a hurdle in the lifeline to HD therapy [8,9]. The role of different cell types, such as endothelial cells (ECs), inflammatory cells, and vascular smooth muscle cells (VSMCs), is being actively investigated, but to date, there is no adequate intervention to promote AVF maturation. Although thoroughly reviewed in models of vascular disease, such as atherosclerosis plaque rupture, aneurysm formation, hypertension, and vascular calcification [10,11,12], the extracellular matrix (ECM) is an active but frequently overlooked participant in the process of AVF maturation, and reviews regarding the role of the ECM in AVF maturation are lacking. As the ECM gives structural and mechanical strength to the vessel and provides communication to the vascular cells to regulate their differentiation and proliferation, it is involved in multiple processes that regulate AVF maturation. The ECM is also essential during the only healthy vascular remodelling comparable to AVF maturation, namely remodelling of the uterine vasculature during pregnancy [13]. Uterine arterial blood flow increases eight-fold over the course of 36 weeks of pregnancy, partially due to ECM-remodelling-induced diameter expansion [13,14].

The importance of the ECM in AVF remodelling is underscored in a recent study by Martinez et al. [15], who performed bulk RNA sequence analysis of pre-access veins and pair-matched AVFs of ESKD patients. They found that ECM proteins were amongst the most differentially expressed genes, with 87% of them upregulated in the AVF. Pathway enrichment analysis showed that collagen remodelling, both degradation and production, was strongly increased. Moreover, clusters of differentially expressed ECM components enabled the crude separation of failed and matured AVFs. These findings demonstrate the importance of ECM remodelling to facilitate increased blood flow in the AVF.

In the present review on the role of the ECM in AVF maturation, AVF failure and the differences in vascular anatomy of arteries and veins will be discussed, followed by the temporal regulation of ECM remodelling post-AVF creation. Factors regulating ECM remodelling will be explored, followed by future perspectives.

## 2. AVF Maturation Failure

As the venous vascular wall differs from the arterial vessel wall, a non-physiological pressure is created in the venous outflow tract. The venous outflow tract thus has to undergo arterialisation: the vessel wall has to thicken to endure the increase in pressure and tensile stress and expand its luminal diameter to facilitate both enhanced blood flow and adapt to the increase in shear stress [16,17]. As this is an intricate process, the venous outflow tract or juxta-anastomotic region of the AVF is often the culprit in AVF maturation and luminal narrowing due to thrombosis and intimal hyperplasia (IH) occurring at the AVF venous outflow tract. IH, together with the degree of enlargement of the vessel diameter, defined as outward remodelling (OR), defines the luminal diameter of the vessel and, thereby, the ability of the AVF to facilitate the increase in blood flow [18]. A disruption in this balance leads to AVF failure, where stenosis decreases the blood flow throughout the AVF and hinders efficient dialysis. To understand the difference between the arterial and venous vessel walls and what happens during AVF remodelling, we will give an overview of the anatomical differences between major arteries and veins and how their mechanical tasks are supported by their differences in the ECM scaffold.

## 3. The Arterial and Venous Vessel Wall and Its ECM Components

### 3.1. Vascular Identity: Phenotypic Differences between Arteries and Veins

Arteries and veins are functionally and anatomically distinct, whereby their arterial-venous cell fate is not only determined by hemodynamic differences but also by genetic mechanisms [19,20,21]. The most well-known arterial and venous markers belong to the Ephrin family [22], which consists of Ephrin ligands and Eph protein-tyrosine kinases receptors. Ligand ephrinB2 marks arterial cells [23], while its receptor Eph-B4 is expressed in venous ECs and VSMCs [24]. Protack et al. [25] showed that murine AVFs adopt a dual arteriovenous identity, with increased expression of both Eph-B4 and Ephrin-B2 in the venous outflow tract shortly after AVF creation. This supports the hypothesis that the venous part of the AVF has to undergo ‘arterialisation’. To understand the process of arterialisation of the AVF, the distinction between the venous and arterial anatomy has to be elucidated.

**Figure 1:**
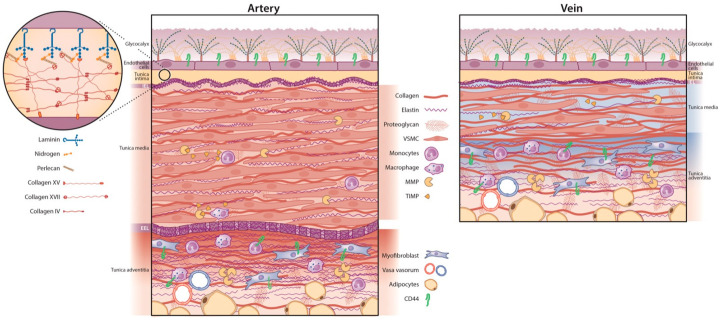
Overview of the arterial versus venous vessel layers and their respective ECM. The luminal side of both vessel types contains an intimal layer with endothelial cells (ECs) covered by the glycocalyx. The basal lamina is a mesh-like structure situated underneath the ECs. The internal elastic lamina (IEL) separates the tunica intima and media. The arterial (**left panel**) tunica media is thicker and more elastic than its venous counterpart (**right panel**) with more vascular smooth muscle cells (VSMCs), organised with elastin into a contractile-elastic unit. Arteries have a prominent external elastic lamina (EEL). Veins are less muscular with a lower elastin-to-collagen ratio. The tunica adventitia contains collagen-producing (myo) fibroblasts, surrounded by perivascular adipose tissue and the vasa vasorum: a capillary network of minor blood vessels. Matrix metalloproteinases (MMPs) are proteinases that regulate ECM degradation and extend into the media and adventitia. They are inhibited by tissue inhibitors of metalloproteinases (TIMPs). CD44 is a cell-surface glycoprotein receptor found in the venous and arterial adventitial layer and venous tunica intima. CD44 is expressed by ECs and can bind ECM components, such as collagen, fibronectin, MMPs, and hyaluronic acid. ECM = extracellular matrix.

AVFs are created from native arteries and veins, which, despite their expressional differences, comprise three layers: the tunica intima, tunica media, and tunica adventitia, sometimes also referred to as tunica externa—three layers with different functions in haemostasis, yet closely working together. The vascular ECM is composed of numerous different macromolecules, proteoglycans, glycoproteins, and cell adhesion proteins that are dispersed throughout the three tunicae and together resist compressive forces, provide adhesive surfaces, and give tensile strength to the vessel. These ECM components are produced by different cell types dispersed throughout the matrix. Figure 1 shows an overview of the anatomy of arteries versus veins and the differences in their ECM structure.

### 3.2. The Tunica Intima

The inner layer of the vasculature is lined by endothelial cells (ECs), which require the ECM for their adhesion, migration, and proliferation. The luminal side of ECs is covered by the glycocalyx: a pericellular matrix composed of glycosaminoglycans (GAGs), proteoglycans, glycolipids, and glycoproteins [26,27]. GAGs, including heparan sulphate and hyaluronan, line the luminal side of the vessel and interact with plasma proteins, chemokines, and growth factors [28]. A healthy glycocalyx is essential for vascular haemostasis as, in addition to interacting with both ECs and plasma-produced molecules, it aids in mechanotransduction, vascular permeability, and, thereby, inflammation [27,29]. The glycocalyx senses and translates mechanical forces into intracellular signals, thereby mediating endothelial nitric oxide (NO) production [30]. Increased shear stress leads to reduced NO, which stimulates heparan sulphate synthesis [31]. In the healthy individual, glycocalyx synthesis and degradation are regulated to maintain endothelial function and adapt to environmental changes. Unfortunately, ESKD patients, and those receiving HD in particular, have elevated serum markers of glycocalyx damage and endothelial dysfunction [32]. This suggests that the vasculature of ESKD patients has reduced capability to adequately sense and respond to a change in shear stress.

In addition to the glycocalyx, ECs also produce and deposit components of the basal lamina and internal elastic lamina (IEL). The basal lamina, also referred to as the basement membrane, is composed of collagens type IV, XV, and XVIII as well as laminin, fibronectin, and perlecan dispersed throughout a mesh-like structure, providing structural support and anchor sites to the ECs [33,34,35]. When the basement membrane is exposed, it forms a bed for activated platelets to adhere [36], which in turn can induce VSMC proliferation and migration to aid in intimal hyperplasia [37,38]. The aforementioned IEL separates the tunica intima from the tunica media.

### 3.3. The Tunica Media

The tunica media is the most prominent vessel layer and highlights the different arterial and venous functions and how their mechanic requirements are translated into their anatomical composition. As arteries are exposed to higher blood pressure, the tunica media is thicker and more elastic than its venous counterpart. Their VSMCs are diagonally organised, whereas venous VSMCs are irregularly organised to make the vessels more dilatable [39].

As indicated by its nomenclature, the IEL is formed by elastin, one of the main components of the vascular ECM. VSMCs produce elastin, proteoglycans, and collagen and disperse these components in between elastic fibres and into the IEL. Elastic fibres are composed of elastin on a microfibril scaffold of fibrillins, microfibril-associated glycoproteins (MAGPs), microfibrillar-associated proteins (MFAPs), and fibulins [40,41]. As VSMCs are dispersed throughout the elastin fibres, they create a contractile-elastic unit and connect adjacent elastic laminae [42]. Veins have lower elastin expression, resulting in reduced transmission of tension throughout the vessel wall and diminished contractability compared to arteries. Arterial biomechanical tasks include dampening pulsation due to cardiac output and blood pressure differences throughout the vasculature [43]. Arterial VSMCs and elastic laminae are organised circumferentially [39], resulting in enhanced elasticity, constriction, and expansion capacity.

Loss of arterial elastin expression increases distensibility both longitudinally and circumferentially [44]. Veins, on the other hand, require less constriction capacity and have a bigger diameter and reduced blood pressure. Therefore, the venous tunica media contains less elastin and more collagen and smooth muscle fibres. Arteries have more abundant elastin expression compared to veins and, thus, a lower collagen-to-elastin ratio [45]. At low pressures, the arterial pressure curve strongly relates to elastin, while at high pressures, the elastic modulus of collagen is more important, indicating the importance of venous collagen deposition after AVF creation [46]. Long-term circumferential strain enhances the expression of both collagen type III and elastin [47]. Collagen limits wall distension by forming a triple helix of elongated fibrils. Collagen type I, III, IV, V, and VI are most abundant in the vasculature and are involved in forming its structure and cell signalling. Fibril-forming collagens—i.e., collagen I, III, and IV—are produced by VSMCs and provide vessel wall strength, as illustrated by the pathology in vascular Ehlers-Danlos syndrome where collagen IIIα1 is mutated, leading to fragile blood vessels and increased incidence of vessel rupture [47,48]. Furthermore, collagens provide binding sites for other ECM components, such as fibronectin and thrombospondin, which regulate vascular remodelling and activity of MMP-2 and MMP-9 [33,49,50].

In vascular homeostasis, the degradation of ECM components is of vital importance to facilitate elastin and collagen turnover. Matrix metalloproteinases (MMPs) produced by VSMCs, monocytes, and macrophages that extend into the media or adventitia are proteinases that partially regulate this ECM degradation [51]. MMPs are specifically inhibited by tissue inhibitors of metalloproteinases (TIMPs). Excessive TIMP production can result in vascular fibrosis, while abundant MMP expression and ECM degradation can lead to aneurysm formation, a form of AVF failure where the vessel wall expands abnormally, resulting in an increased risk of vessel rupture [52]. The formation of both elastin and collagen is dependent on lysyl oxidase (LOX), as LOX induces extracellular catalysis of lysine and hydroxylysine residues for collagen and elastin cross-linking [53,54]. Interventions in elastin and collagen production and turnover to promote AVF functioning will be discussed later.

### 3.4. The Tunica Adventitia

The tunica adventitia is separated from the tunica media by the external elastic lamina (EEL). Overall, the adventitia of large vessels is characterised by collagen-producing (myo)fibroblasts forming fibrous connective tissue, which is surrounded by perivascular adipose tissue (PVAT) and the vasa vasorum: a capillary network of minor blood vessels supplying major blood vessels with oxygen and nutrients [33,55]. Increased vasa vasorum is often observed in pathological processes [33]. In major arteries, the vasa vasorum spans from the adventitia to the outer layer of the media, while in large muscular veins, the vasa vasorum extends deeply into the tunica media [56]. However, vascularisation of the pre-access vein or venous AVF outflow tract is not associated with maturation outcomes [57].

Myofibroblasts in the tunica adventitia are major collagen producers, which they secrete into the tunica media to aggregate into fibrils to provide mechanical strength and prevent extreme vasodilation. The majority of the adventitial ECM is formed by elastin, fibronectin, proteoglycans, and collagen type I to prevent vessel rupture. Moreover, it serves as storage for molecules such as growth factors and MMPs. CD44 is a cell-surface glycoprotein receptor found in the venous and arterial adventitial layer and venous tunica intima [58]. CD44 is expressed by inflammatory and vascular cells such as ECs and fibroblasts, which can bind ECM components, such as collagen, fibronectin, MMPs, and hyaluronic acid [59]. As the adventitia contains ECM adhesion proteins such as CD44 and fibroblasts, which produce a majority of vascular ECM components, injury of the adventitia can radically alter the vascular ECM [33,60].

## 4. The Effect of Chronic Kidney Disease on the Vasculature

Kidney failure and cardiovascular pathology often go hand in hand, and reduced glomerular filtration rate (GFR) is an independent risk factor for cardiovascular events [61]. When creating an AVF, patients are at the ESKD stage; thus, the vessels are affected by an uremic environment and exposed to high blood pressure, as the majority of ESKD patients have hypertension. These factors affect vascular health and contribute to arterial stiffening, vascular calcification, increased risk of atherosclerosis, and impaired vascular repair [62]. Wali et al. [63] described that cephalic veins of ESKD patients show morphologically altered VSMCs and increased irregular deposition of collagen and elastin in between VSMCs when compared to healthy controls. Native veins of ESKD patients pre-AVF surgery contain significant collagen deposition in the tunica media and an enlarged intima mostly consisting of proteoglycans and collagen [64]. Furthermore, there is increased phagocytic activity of elastin and collagen in medial VSMCs [63]. Kidney failure is often paired with endothelial dysfunction, increased oxidative stress, VSMC proliferation, and peripheral vascular dysfunction [65,66,67]. Previous research in rats with kidney failure revealed that the increased oxidative stress and enhanced NO resistance hinder AVF maturation [68]. It has been shown that antioxidant selenium can modulate oxidative stress in CKD patients and reduce inflammation and oxidative stress markers [69].

However, when studying the vascular remodelling of AVFs, it is important to keep in mind that kidney failure impacts vascular health.

## 5. ECM Remodelling in the AVF: A Timely Matter

AVF maturation research is mostly focused on the venous outflow tract and its adaptation to the newly created arterial environment. Recently, there has been an increasing interest in modulating the ECM to promote AVF maturation and functionality. Hall et al. [70] elegantly studied temporal regulation of venous ECM remodelling after AVF creation in an aortocaval AVF model in C57BL/6J mice.

Murine AVF maturation encompasses three distinct phases of ECM remodelling: early ECM degradation, followed by a transition phase through reorganisation of the collagen and elastin scaffold, and lastly, rebuilding of the matrix with non-collagenous proteins and glycoproteins. Hence, timely expression of factors regulating the ECM is especially important after AVF creation.

Early MMP expression was observed in murine AVFs that would mature: *MMP-9* mRNA was increased maximally on day 1, while *MMP-2* RNA and protein expression increased on day 7 [70]. *TIMP1* RNA expression was also elevated on day 1 until 21 days post-AVF creation, whereas expression of *TIMP2*, *TIMP3* and *TIMP4* was upregulated later in the remodelling process at day 21. *Collagen III* mRNA showed upregulation on day 3 post-AVF creation, while *collagen I*, *IV*, *VIII*, and *XVIII* showed increased expression on day 7. Collagen III deposition was mainly observed in the adventitia and collagen I within the layers of the remodelling venous outflow tract. Total collagen protein expression was upregulated at day 21. *Elastin* mRNA expression was increased at 3 and 7 days post-AVF creation, and elastin protein deposition was elevated at day 28.

**Figure 2:**
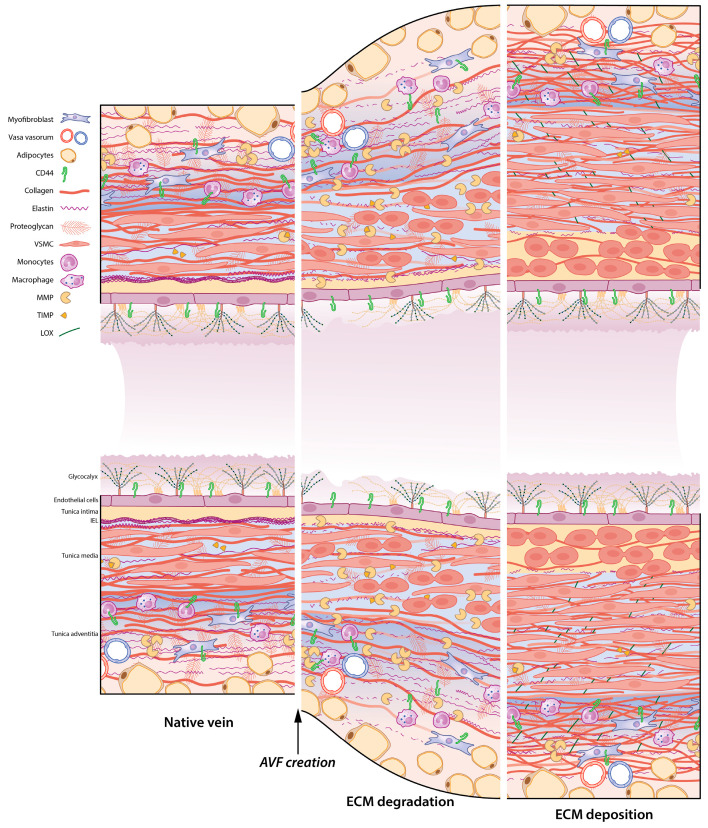
ECM remodelling after AVF creation. The native vein (**left panel**) requires rebuilding its vascular scaffold to facilitate AVF maturation. The venous outflow tract undergoes ECM degradation (**middle panel**) and rebuilding of the ECM framework through ECM deposition (**right panel**). After AVF creation, the intimal layer and glycocalyx are damaged, and increased wall shear stress induces outward remodelling. Degradation of the ECM is facilitated by increased MMP (matrix metalloprotease) production, degrading collagen and elastin. Proliferating VSMCs ensure outward remodelling and wall thickening. Eventually, VSMCs migrate into the intima, where they proliferate and form intimal hyperplasia. During ECM deposition, collagen is produced by VSMCs and myofibroblasts. LOX (lysyl oxidase) cross-links elastin and collagen into their respective fibre formations.

As MMP expression is regulated post-transcriptionally [71], data about mRNA expression should be interpreted with caution. However, the work of Hall et al. [70] indicates that the balance between vessel wall degradation and remodelling post-AVF creation is a fine line. Figure 2 gives a timeline overview of balanced degradation and reconstruction of the ECM after AVF creation. Next, studies on the ECM in AVF maturation will be discussed in the sequence of events occurring in AVF vascular remodelling: (i) ECM degradation regulated by MMPs and TIMPs, (ii) regulation of collagen and elastin deposition and LOX and (iii) the effects of TGF-β and inflammation on ECM remodelling.

## 6. Rebuilding the Vascular Framework: ECM Remodelling during AVF Maturation

### 6.1. ECM Degradation: The Role of MMPs and TIMPs in the AVF

ECM degradation, due to MMP activation or TIMP inhibition, is an essential process early on in AVF maturation. Rat AVFs showed an increase in both blood flow and intimal and medial area post-AVF creation compared to sham-operated controls, accompanied by an increase in MMP-2 and MMP-9 and downregulation of TIMP-4, which resulted in collagen degradation and an increased collagen I/III ratio [72]. At the time of AVF creation, elevated serum levels of MMP-2/TIMP-2 are measured in patients that would have a matured AVF compared to those who would experience AVF failure, with border-significant elevated levels of MMP-9/TIMP-4 as well (*p* = 0.06) [73]. Another study verified that patients with veins that would become matured AVFs had increased pre-operative expression of TIMP-2, MMP-2 activator MT1-MMP (Membrane type-1-MMP), and MMP-2 itself when compared to veins that would become failed AVFs [74]. Misra et al. [75] show that in rats, increased expression of MMP-2 and MMP-9 at a later time point is associated with venous stenosis, similar to ESKD patient data on increased MMP-9 expression in stenotic AVF lesions [76]. This is in line with the increased deposition of pro-MMP-9 in AVFs that had to undergo surgical revision due to thrombosis or stenosis [77]. Many clinical descriptive studies have focused on the role of MMPs in AVF vascular remodelling but encompass findings based on systemic plasma levels or patient tissue of AVFs that need to undergo revision and are, thus, AVFs that failed to mature. This raises the question: what is the role of MMPs and TIMPs locally in AVF maturation? Therefore, interventions in MMP and TIMP expression are essential to see if they could be a therapeutic target, and what the therapeutic timing should be.

Doxycycline has been used as a specific MMP inhibitor as it directly inactivates MMPs through their zinc sites and indirectly through binding to inactive calcium sites [78]. Nath et al. [79] administered doxycycline chronically in their murine carotid-jugular AVF model. They verified suppression of MMP-9 expression in the venous outflow tract of the AVF, which did not affect AVF patency. Nonetheless, doxycycline is proven effective in preventing IH in a mouse model of arterial intimal hyperplasia and vein graft thickening [80]. Conditional forward logistical analysis was used with patients undergoing chronic maintenance haemodialysis to assess the likelihood of vascular access aneurysm formation after doxycycline treatment. Here, doxycycline treatment was compared to other antibiotic treatments. Patients that had received doxycycline and thus MMP inhibition appeared to have a decrease in aneurysm formation: a long-outcome effect rather than short-term maturation [78].

Shih et al. [81] studied the direct effect of MMP-9 deficiency on AVF remodelling using a murine knockout model with chronic kidney injury. MMP-9 deficient mice showed reduced VSMCs and collagen, accompanied by an increase in the AVF-venous luminal area and a decrease in IH when compared to wild-type (WT) mice. This was due to a reduction in vascular inflammation, demonstrated by reduced CD44 protein expression. After a femoral artery wire injury, MMP-9 and MMP-2 deficient mice showed reduced IH formation at two weeks post-injury and at four weeks post-injury [82]. As for patients studies, Lin et al. [83] state that the variant and accompanying transcriptional activity of MMPs is associated with AVF outcome in patients: genetic variants that lead to reduced transcription of MMP-1, MMP-3 and MMP-9 are associated with increased risk of AVF failure and stenosis, probably due to reduced proteolytic degradation of the ECM, resulting in ECM accumulation. Taken together, there is a solid foundation to state that MMPs play a significant role in AVF remodelling in animal models, which can possibly be translated to patients.

### 6.2. Strengthening the ECM Framework: Macro-Proteins Elastin and Collagen

**Figure 3:**
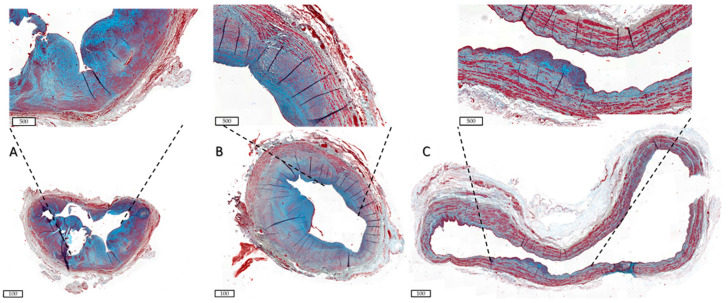
Differential ECM deposition in human AVFs. Samples were stained with Masson Trichrome. ECM is shown in blue, and smooth muscle cells are stained red. Scale bars are 100 µm in the bottom images and 500 µm in the inlay. (**A**) shows an AVF with little outward remodelling and a lot of ECM deposition in the IH, as shown in the inlay, indicative of a fibrotic AVF. (**B**) AVF with wall thickening and some ECM deposition amongst well-organised muscle fibres. (**C**) AVF with excessive OR and little ECM deposition, indicative of aneurysm formation. AVF = arteriovenous fistula, ECM = extracellular matrix, IH = intimal hyperplasia, OR = outward remodelling.

Collagen and elastin are major ECM components that give structure to the vessel. Vascular pathologies emphasise the importance of the maintenance of these ECM proteins. Loss of both elastin-network integrity, as seen in Marfan syndrome [40,84], and loss-of-function mutations in collagen [48,85], both result in a weakened vessel wall and increased aneurysm formation. Excessive collagen deposition, however, is also a common cause of vascular clinical manifestations, seen in vessel fibrosis and vascular stiffness [86]. Figure 3 shows patient AVFs with different gradients of ECM deposition.

It has been established that mechanical factors, such as the increase in flow and wall shear stress stretching the elastic fibres, can increase the binding of elastase and the density of its binding sites along the fibre [87]. After AVF surgery, degeneration of the internal elastic lamina indeed occurs both arterially and at sites of the venous outflow tract that experience the biggest hemodynamic changes proximal to the anastomosis [88,89,90]. Rabbit AVF models show flow and shear stress-induced elastin fragmentation post AVF creation in the afferent artery [90,91,92,93,94], preceded by increased mRNA expression of both MMP-2, MMP-9, and MT1-MMP in ECs and VSMCs [92]. These fragmentations of the IEL contain VMSC deposition in between the laminae, facilitating OR and, thus, luminal enlargement [90,94]. Elastin haplodeficiency in a murine carotid-jugular AVF model resulted in accelerated OR of the venous outflow tract, no differential development of IH, and, eventually, an increase in the AVF venous luminal area [95]. Meanwhile, increased elastin deposition and a reduction in elastase activity hinder elastin degradation and impair OR and murine AVF maturation [96].

Two recent randomised, double-blind, placebo-controlled interventions using recombinant human elastase PRT-201 were performed in patients receiving a brachio- or radiocephalic AVF [97,98]. In these studies, PRT-201 was applied to the inflow artery, anastomosis, and outflow vein. Usage was proven safe, and there was no increase in adverse events; however, there was no increase in AVF venous diameter, stenosis, blood flow, or successful maturation compared to placebo-treated patients. PRT-201 was also not proven effective in AVGs [99]. Low-dosage PRT-201 was associated with improved unassisted maturation [100]. A larger prospective trial again showed the safety of using PRT-201 but no clinical value to surgical outcomes of radiocephalic fistulas nor secondary patency [97]. This indicates that increasing elastin degradation through temporary supplementation of an elastase might not be of therapeutic value to improve the maturation and patency of AVFs. It is hypothesised that the degradation of elastin has to be guided by the repair of elastic fibres [70,101].

Case reports of Alport’s syndrome patients, a renal disease caused by a systemic disorder of collagen type IV (COL4A5), show a high prevalence of aneurysmal AVFs [102,103]. An increase in collagen content in the venous AVF is essential [104]; however, excessive collagen deposition leads to fibrosis. Interestingly, pre-existing arterial medial fibrosis is positively associated with AVF diameter, blood flow, and AVF maturation, while pre-existing venous medial fibrosis did not correlate with AVF functionality [105]. Excessive fibrotic remodelling in the form of circumferential alignment of collagenous fibres along the venous AVF outflow tract is associated with the non-maturation of brachiobasilic AVFs [106]. Bulk RNA sequence analysis of pre-access veins and pair-matched two-stage brachiobasilic AVFs of ESKD patients showed increased expression levels of fibrillar collagens I and II in the AVF, whereby increased COL8A1 and decreased MMP-9 and MMP-19 expression marked failed AVFs [15]. Upregulated COL8A1 results in increased collagen VIII, which forms a hexagonal network instead of fibres surrounding VSMCs in the tunica media. Martinez et al. hypothesise that the increase of collagen VIII in the medial layer might induce enhanced TGF-β and collagen I and collagen III production, resulting in vascular stiffness and AVF failure [15].

Little is known about the effect of collagen modulation post-AVF creation, as only a few interventional studies have been performed. Inhibiting LOX is the most common method used to study the role of collagen expression in vascular remodelling post-AVF, as it aids in intra- and intermolecular covalent cross-linking of collagen and facilitates the formation of collagen’s triple helix conformation [107]. This is often performed by administering β-aminopropionitrile (BAPN), an irreversible LOX inhibitor. Hernandez et al. [108] inhibited LOX both locally and systemically through intraperitoneal BAPN injection or a BAPN-loaded scaffold around the venous outflow tract of an end-to-side rat AVF of the epigastric vein and femoral artery. The scaffold was degraded in 60 days, and systemic delivery was given from 2 days prior to 21 days post-AVF creation. Both treatments decreased fibrosis and shear stress and improved flow volume and distensibility in the AVF. A follow-up rat study showed that a BAPN-loaded PGLA nanofibre scaffold around the AVF promotes OR, prevents adventitial fibrosis, and improves vascular compliance [109]. BAPN and LOX, however, are both involved in cross-linking of collagen as well as elastin fibres and are, therefore, not exclusively related to collagen [110]. In ESKD patients, pre-access veins that would fail had higher LOX expression compared to veins that would mature. In failed AVFs, increased collagen cross-linking was observed [108]. Thus, modulation of LOX activity, and, thereby, elastin and collagen, seems to be a promising therapeutic target. However, as we can conclude from clinical trials administering PRT-201, interfering with elastin fragmentation is a delicate matter in AVF remodelling. Similar to the modulation of MMP and TIMP activity, inducing collagen and elastin degradation to promote AVF functionality is a timely matter and should be balanced by a certain degree of ECM production, orchestrated into a balanced sequence of events.

### 6.3. The Effect of TGF-β on ECM Remodelling in the AVF

In addition to regulating elastin and collagen cross-linking, LOX also influences the activation of the TGF-β pathway. TGF-β can be activated through the canonical pathway (Smad-activation) or non-canonical pathway (non-Smad-regulated) and generally results in myofibroblast activation, ECM production, and prevention of ECM degradation [111]. LOX suppresses Smad3 phosphorylation, a signalling intermediate normally activated by TGF-β stimulation to induce VSMC proliferation [112]. TGF-β, in turn, enhances LOX expression in rat aortic VSMCs [113,114]. TGF-β has been proven a key player in both kidney and vascular fibrosis and an important determinant for AVF functioning, as demonstrated by interventional in vivo studies summarised here.

Inhibiting TGF-β receptor-I through adventitial administration of SB431542-loaded nanoparticles reduces AVF wall thickness, collagen deposition, and VSMC proliferation [115]. Cell-specific TGF-β inhibition in ECs or VSMCs results in a reduction of collagen density, and EC-specific modulation of TGF-β signalling affects wall thickness, OR, and AVF patency [115]. Murine and matured patient AVFs show non-canonical activation of TGF-β signalling through TGF-β-activated kinase 1 (TAK1): reducing TAK1 function from 7 days prior to 7 days post-surgery using 5Z-7-oxozeaenol (OZ) results in decreased fibronectin, collagen I, wall thickness, and vessel diameter, which are increased when overexpressing TAK1 through periadventitial lentiviral transduction [116]. Differential regulation of TGF-β signalling might also underly the sex difference that is observed in AVF maturation, where female ESKD patients have decreased AVF maturation and patency [117,118]. Cai et al. [119] observed decreased gene expression of *BMP7* and *IL17Rb* and increased *Tgf-β1* and *Tgfβ-r1* in female murine AVFs, which resulted in venous fibrosis and negative vascular remodelling. Percutaneous transluminal angioplasty (PTA), a common intervention to salvage AVF functionality, increased TGF-β signalling in female but not male murine AVFs. This resulted in a reduction in the diameter of the venous outflow tract, luminal area, peak systolic velocity and an increased intima-to-media ratio [120]. In addition to murine studies, TGF-β is shown to play a role in human AVF maturation as well. Heine et al. [121] observed that polymorphisms that cause increased TGF-β1 expression were associated with a decreased 12-month patency rate of 62.4%, while intermediate production of TGF-β1 was associated with a patency of 81.2%. More research is needed to evaluate if TGF-β modulation is a valuable therapeutic remedy to reduce fibrosis and collagen deposition in the AVF.

### 6.4. Inflammation Influencing ECM Remodelling

Inflammation is an inherent process of AVF creation. Several inflammatory markers have been related to poor AVF patency outcome or AVF complications, including elevated systemic C-reactive protein (CRP) levels, neutrophil-to-lymphocyte ratio (NLR), platelet-to-lymphocyte ratio (PLR) and systemic inflammatory index (SII) as well as the local deposition of IL-6, TNF-α, and MCP-1 in the AVF [122,123,124,125,126,127]. Administering liposomal prednisolone inhibited inflammatory markers IL-6, TNF-α, and MCP-1 in a murine AVF model [128]. Liposomal prednisolone was proven safe in humans but has not been studied yet in a large randomised control trial [129]. Similar to ECM remodelling, inflammation requires a balanced approach between pro-inflammatory and anti-inflammatory signalling to facilitate OR and wall thickening. The influence of inflammation on AVF vascular remodelling is thoroughly reviewed [130,131,132,133,134,135].

Here, we uncover the relationship between inflammation and ECM remodelling in an AVF model, whereby inflammation can increase both ECM degradation and deposition. In the context of this review, we broadly uncover two categories in the relationship between inflammation and ECM remodelling in the AVF. Namely, inflammation-influencing MMP production by VSMCs and macrophages and inflammation-enhancing CD44 activity.

Inflammatory cells, such as macrophages and T-cells, produce numerous factors and cytokines that affect the vascular ECM, such as TGF-β and MMPs, by regulatory T cells and macrophages [136,137]. Pro-inflammatory M1 macrophages secrete pro-inflammatory cytokines such as TNF-α, IFN-γ, and IL-1 [134]. These local inflammatory stimuli can enhance regional upregulation of MMPs, induce elastin breaks, and thereby facilitate VSMC migration and degradation of the vascular ECM structure. IL-1 or TNF-α stimulation of VSMCs can lead to MMP-1, 3, and 9 production, but not TIMPs, and IL-1 is essential to facilitate OR [138,139]. Thrombosed AVFs are characterised by increased inflammation at the luminal site through MMP-9 expressing macrophages [124]. Inflammatory stimuli through CD44 activation, however, lead to the upregulation of M2 macrophages, which produce IL-10 and TGF-β, inducing ECM deposition, and leading to venous wall thickening, thereby promoting AVF maturation [134,140]. Administering cyclosporine, a T-cell inhibitor, resulted in reduced pro and anti-inflammatory macrophage presence in AVFs, hindering wall thickening, and promoting OR [141]. The relationship between inflammation-induced ECM remodelling, OR, and wall thickening versus IH formation due to VSMC proliferation and thrombi is complex and temporal and needs to be unravelled further to design therapeutic agents for AVF maturation.

## 7. Interventions Creating an ECM Framework Supporting Arteriovenous Fistulas

A few interventional studies have been performed in animal models using an ECM-mimicking framework to wrap around the AVF and possibly enhance AVF maturation.

CorMatrix, a decellularised matrix containing mostly collagen I fibres, GAGs, and glycoproteins, was wrapped around the venous outflow tract of carotid-jugular AVFs in immunodeficient mice. CorMatrix had beneficial outcomes regarding luminal outflow area and OR [142]. It is hypothesised that CorMatrix traps adventitial fibroblasts and prevents their differentiation and inwards migration, thereby reducing IH. CorMatrix has been applied in patient AVFs that needed reconstruction, and the data suggests that CorMatrix is safe to use, yet a high incidence of IH occurred, and there was no control group [143]. One case report shows the use of CorMatrix to repair an aneurysmal AVF, which resulted in complication-free clinical patency for the duration of a follow-up of four months [144]. A clinical study, however, has to be performed to investigate the effectiveness of the CorMatrix application peri-operatively. Natural Vascular Scaffolding (NVS) Therapy is another ECM intervention studied in rats. A small molecule (4-amino-1,8-naphtalamide) was administrated perivascular at the AVF anastomosis and activated with a laser, which induces covalent binding of collagen and elastin, resulting in a framework for the vascular wall [145]. This resulted in an increase in AVF luminal area compared to controls, with decreased MMP-2 and MMP-9 expression at four weeks. Less collagen deposition was observed, although not significant, and the collagen fibres were organised more perpendicular [145], which seems favourable in AVFs [106]. The same treatment was also tested in sheep after cephalic veins were dilated through a balloon catheter [146]. This led to an increase in wall thickness and luminal diameter. These ECM interventions applying or creating an ECM-wrap peri-operatively during AVF surgery in animal models suggest that these remedies are safe and may have therapeutic potential by facilitating OR and luminal expansion to enhance AVF maturation.

## 8. Future Directions

In this review, we have summarised the influence of the ECM in AVF remodelling. Restructuring the ECM after AVF creation is a complex and timely process, with a dynamic balance between ECM degradation and ECM synthesis, mostly occurring in the venous tract. ECM degradation is essential for OR and venous wall thickening. However, excessive ECM degradation or insufficient repair results in a weak aneurysmatic venous outflow tract. Most novel therapeutic interventions focused on the ECM have solely been studied in animal models of AVF failure, and the success of those interventions is primarily based on (end-point) histological and morphometric parameters. To unravel how timely ECM turnover influences AVF outcome, new research methods should be employed to study ECM turnover longitudinally.

Currently, there are several live-imaging modalities available to track AVF flow, ECM remodelling and wall thickening in vivo, including magnetic resonance imaging (MRI), ultrasound analysis (US) and photoacoustics. Increased MRI specificity can be achieved through the use of contrast agents, for example, targeting elastin or tropoelastin [84,147,148]. US analysis can both determine AVF flow [149] and track wall thickening, total vessel wall area and OR [150,151]. By determining the optical absorption contrast of the target area, photoacoustics can characterise different tissues and distinguish collagen from its surroundings [152]. Live tissue imaging can also be accompanied by labelling agents targeting collagen [153,154,155]. MMPSense is a protease-activatable fluorescent agent activated by MMP-2, -3, -9, -12, and 13, which allows real-time imaging of local MMP activation and, thereby, ECM turnover [156]. These imaging techniques are suitable for studying ECM turnover post-AVF creation in vivo and should be incorporated into future research.

Furthermore, it is important to approach vascular AVF remodelling as a system tightly regulated by both vascular cells and the ECM that they produce. AVF maturation is an interplay between regulated inflammation and non-excessive cell proliferation to facilitate re-endothelisation, wall thickening, and OR and ECM turnover. Therapeutic interventions should, therefore, have time-dependent delivery. Post-surgery, peri-vascular scaffolds can easily be applied when constructing an AVF to target early vascular remodelling. During this timeframe, OR is the most prominent process, while IH develops at a later time point. The scaffolds could contain slow-release gels, anti-inflammatory cytokines, small molecules, or gene therapy. Such an example has already been described above, with Natural Vascular Scaffolding Therapy as an ECM intervention that interlinks collagen and elastin via photoactivation of a locally delivered small molecule (4-amino-1,8-naphtalamide) [145]. Another small molecule that showed promise in preventing AVF failure is MCC950. This NLRP3 inflammasome inhibitor was shown to repress Smad2/3 phosphorylation and suppress CKD–promoted AVF failure [157]. Another study showed that AVF failure due to IH and subsequent venous stenosis in a porcine AVF model could be improved by using 1α,25(OH)2D3-encapsulated nanoparticles. Perivascular AVF release of this molecule from poly(lactic-co-glycolic acid) nanoparticles that were embedded in a pluronic F127 hydrogel resulted in better AVF flow and hemodynamics, while it also reduced inflammation and fibrosis [158]. Vascular scaffolds could also be implemented to study the effect of redox regulation on ECM components. Both cross-linking and microfibril-assembly of collagen, elastin, and fibrillin are known to be influenced by intrinsic redox regulation, affecting the protein’s cysteine residues and MMP-2 and MMP-9 [159]. Moreover, adenoviral-delivered gene therapy is a well-studied delivery method in vein grafts and AVFs [160,161,162] and could be employed to target LOX, MMPs, or TIMPs.

An alternative approach can be found in the capacity of non-coding RNA, such as microRNAs (miRNAs), to post-transcriptionally regulate ECM composition and AVF outcome. For instance, miR-29a/b regulates ECM mRNAs, such as collagen (type I and II), fibronectin, and elastin, and inhibits their translation [163]. As such, miR-29 inhibitors can stimulate elastin formation and LOX expression [164,165], although this could also induce a fibrotic response and vascular calcification [163,166]. Possibly, combination therapy with LOX-inhibition might achieve synergistic favourable effects on AVF maturation, especially when locally applied and in a timely manner. Interestingly, miR-21 also directly affects the synthesis of several collagen species, and miR-21 inhibition improved AVF patency by reducing IH and VSMCs and myofibroblast presence in the vessel wall [167].

In conclusion, the ECM is a vital scaffold for vessels, and its remodelling post-AVF creation is a balance between degradation and deposition, enabling outward remodelling of the AVF, followed by supporting and strengthening it. This requires timely therapeutic delivery, taking into account the interplay between the vascular cells and the ECM they both produce and reside in.

## Data Availability

Not applicable.
